# Aufbau des neuen „Forschungsdatenzentrums Gesundheit“ zur Datenbereitstellung für die Wissenschaft

**DOI:** 10.1007/s00103-023-03831-z

**Published:** 2024-01-12

**Authors:** Melanie Ludwig, Katharina Schneider, Steffen Heß, Karl Broich

**Affiliations:** 1 Forschungsdatenzentrum, Bundesinstitut für Arzneimittel und Medizinprodukte (BfArM), Kurt-Georg-Kiesinger Allee 3, 53175 Bonn, Deutschland; 2 Bundesinstitut für Arzneimittel und Medizinprodukte (BfArM), Kurt-Georg-Kiesinger-Allee 3, 53175 Bonn, Deutschland

**Keywords:** Sekundärdatenanalysen, Routinedaten, Versorgungsqualität, Datenschutz, Medizinforschung, Secondary data analysis, Routine data, Quality of care, Data protection, Medical research

## Abstract

Die Analyse von Real-world-Daten (RWD) hat in der Gesundheitsforschung der vergangenen Jahre eine immer größere Bedeutung gewonnen. Mit dem im Aufbau befindlichen Forschungsdatenzentrum Gesundheit („FDZ Gesundheit“) werden Forschende künftig Zugang zu Routinedaten der gesetzlichen Krankenversicherungen von rund 74 Mio. Menschen in Deutschland erhalten können. Auch Daten aus den elektronischen Patientenakten können hier mit der Zeit für die Forschung bereitgestellt werden. Dabei gewährleistet das FDZ Gesundheit höchste Datenschutz- und IT-Sicherheitsstandards. Der digitale Antragsprozess, die Datenbereitstellungen in sicheren virtuellen Analyseräumen sowie die Features zur Unterstützung der Auswertungen, wie Kataloge von Kodiersystemen, ein Point-and-Click-Analysetool und vordefinierte Standardauswertungen, erhöhen die Nutzungsfreundlichkeit für die Forschenden. Durch die Analysen der umfangreichen im FDZ verfügbaren Gesundheitsdaten ergeben sich zukünftig vielfältige Möglichkeiten, das Gesundheitssystem und die Versorgungsqualität zu verbessern.

In diesem Beitrag werden zunächst die Vorteile des FDZ Gesundheit beleuchtet und die sich durch das FDZ ergebenden Möglichkeiten für die Forschung in der Versorgung und für die Bevölkerung exemplarisch skizziert. Danach werden die Struktur und die zentralen Aspekte des FDZ Gesundheit erläutert. Ein Ausblick auf die Chancen der Verknüpfung verschiedener Daten wird gegeben. Wie die Antrags- und Datennutzungsprozesse am FDZ aussehen werden, wird dabei am Beispiel von fiktiven Möglichkeiten zur Analyse von Long COVID anhand der künftig verfügbaren Abrechnungsdaten dargestellt.

## Abstract (en)

The analysis of real-world data (RWD) has become increasingly important in health research in recent years. With the BfArM Health Data Lab (HDL), which is currently being set up, researchers will in future be able to gain access to routine data from the statutory health insurance of around 74 million people in Germany. Data from electronic patient records can also be made available for research prospectively. In doing so, the Health Data Lab guarantees the highest data protection and IT security standards. The digital application process, the provision of data in secure processing environments as well as the features supporting the analyses such as catalogues of coding systems, a point-and-click analysis tool and predefined standard analyses increase user-friendliness for researchers. The use of the extensive health data accessible at HDL will open a wide range of future possibilities for improving the health system and the quality of care. This article begins by highlighting the advantages of the HDL and outlining the opportunities that the RWD offers for research in healthcare and for the population. The structure and central aspects of the HDL are explained afterwards. An outlook on the opportunities of linking different data is given. What the application and data usage processes at the HDL will look like is illustrated using the example of fictitious possibilities for analysing long COVID based on the routine data available at the HDL in the future.

## Hintergrund

Die Analyse von Real-world-Daten (RWD) im Rahmen von Sekundärdatenanalysen gewinnt in der heutigen Zeit mehr und mehr an Bedeutung. Das Forschungsdatenzentrum Gesundheit („FDZ Gesundheit“) dient dem Zweck, künftig die Routinedaten der gesetzlichen Krankenversicherungen von rund 74 Mio. Menschen in Deutschland für die Forschung bereitzustellen.

Im Folgenden werden zunächst die Vorteile des FDZ Gesundheit anhand des Beispiels von Analysen zum Long-COVID-Syndrom beleuchtet und die sich durch das FDZ ergebenden Möglichkeiten und Chancen für die Forschung in der Versorgung und für die Bevölkerung exemplarisch skizziert. Im Anschluss werden die Struktur und die zentralen Aspekte des sich im Aufbau befindlichen FDZ Gesundheit erläutert. Informationen darüber, wann eine Antragsstellung möglich ist, werden rechtzeitig auf der Internetpräsenz des FDZ Gesundheit bereitgestellt.^1^

## Chancen von Real-world-Daten am Beispiel Long COVID

Drei Jahre lang hatte die COVID-19-Pandemie die Welt fest in ihrer Hand. Deutlich hat sich in dieser Zeit gezeigt, dass die Nutzung von Sekundärdaten im Gesundheitswesen immense Möglichkeiten bietet. Insbesondere für die Forschung zu den Spät- und Langzeitfolgen einer COVID-19-Erkrankung (Long COVID) können Sekundärdaten einen wertvollen Beitrag leisten. Long COVID betrifft schätzungsweise 65 Mio. Menschen weltweit, die weiterhin von den Folgen der Infektion mit SARS-CoV‑2 betroffen sind [1]. Diese Menschen warten auf wichtige Forschungsergebnisse, die eine evidenzbasierte Therapie ihrer Erkrankung ermöglichen und ihre Versorgung verbessern. So betonte Gesundheitsminister Prof. Dr. Karl Lauterbach im Juli 2023 in einer Pressekonferenz: „Für Menschen mit Long COVID ist die Pandemie leider noch nicht beendet, sie leiden immer noch unter den Folgen, warten auf Forschungsergebnisse, Therapien, gute Versorgung. (…) Der Schrecken der Pandemie ist Vergangenheit. Die Langzeitfolgen bleiben eine Herausforderung. Die Long-COVID-Kranken erwarten zu Recht, dass wir uns um sie kümmern.“^2^

Viele Forschende haben sich zum Ziel gesetzt, mögliche Ursachen und Therapieansätze für Long COVID zu erforschen. Um dieses Vorhaben umzusetzen, benötigen sie eine große Menge an Daten, und das möglichst schnell. Die Durchführung von Studien ist aufwändig, zeit- und kostenintensiv. Zudem stellt die unterschiedliche Schwere der Verläufe bei Long-COVID-Betroffenen eine zusätzliche Herausforderung für ein geeignetes Studiendesign dar. Etwa 5 % dieser Erkrankten sind sehr schwer betroffen, was über 3 Mio. Menschen weltweit entspricht [2]. Eine vielversprechende Möglichkeit eröffnet sich in solchen Fällen durch anderweitig erfasste Gesundheitsdaten in Form von Sekundärdaten. Die Analyse von Real-world-Daten (RWD) bzw. konkret der in der Krankenversorgung entstehenden Daten kann eine hilfreiche und wertvolle Ergänzung zum „Goldstandard“ der klinischen Studien darstellen.

Mit dem Aufbau des FDZ Gesundheit wird sich die Nutzung von RWD deutlich verbessern und ein geeigneter Zugang zu solchen Daten kann schneller erfolgen. Künftig werden im FDZ Gesundheit eine große Bandbreite der Abrechnungsdaten aller gesetzlich versicherten Menschen in Deutschland sowie perspektivisch spezifische Daten aus der elektronischen Patientenakte für Forschungsfragen zur Verfügung stehen. Ein Einblick in Art und Umfang der Sekundärdaten ist im nachfolgenden Kapitel dargestellt. Dabei unterliegt die Analyse von Sekundärdaten verschiedenen Herausforderungen und auch deren Herkunft ist bei der Interpretation einer Analyse zu beachten. Hierfür sei auf gängige Literatur wie z. B. die „Gute Praxis Sekundärdatenanalyse“ [3] verwiesen.

Insgesamt wird die gesamte Bevölkerung von den Studien profitieren, die durch die Datennutzung über das FDZ Gesundheit ermöglicht werden können. Neben den Potenzialen einer besseren Versorgung können durch die Analyse der im FDZ verfügbaren Daten Forschungsvorhaben durchgeführt werden, anhand derer Risikofaktoren und Frühwarnzeichen für bestimmte Krankheiten frühzeitig identifiziert werden können. Durch rechtzeitige Interventionen und Vorsorgemaßnahmen können Krankheiten möglicherweise verhindert oder in einem frühen Stadium erkannt und behandelt werden, was zu besseren Behandlungsergebnissen und einer insgesamt verbesserten Gesundheit beitragen kann. Insbesondere ermöglicht die Analyse großer Datenmengen im FDZ, individuelle Unterschiede in der Wirksamkeit von Behandlungen und Medikamenten besser zu verstehen. Dadurch können Informationen über die Wirksamkeit und Sicherheit von Medikamenten und Therapien gesammelt werden, die das Wissen aus klinischen Studien ergänzen. Diese Erkenntnisse können auf lange Sicht potenziell zur Verbesserung der Arzneimittelsicherheit und der frühzeitigen Erkennung von Risiken und Nebenwirkungen beitragen. Diese Potenziale werden auch in der Arzneimittelregulation gesehen, sodass zur Stärkung der Rolle der Real-world-evidence vom European Medicines Regulatory Network (EMRN) eine entsprechende Strategie entwickelt und veröffentlicht wurde [4].

Forschungsergebnisse, die in Zukunft über Studien mit den Daten des FDZ Gesundheit entstehen werden, können dann indirekt auch Versorger:innen wie Ärzt:innen in ihrer täglichen Arbeit unterstützen. Neue Erkenntnisse und evidenzbasierte Informationen können dazu beitragen, fundierte Entscheidungen über Diagnosen, Behandlungen und Therapiepläne zu treffen. Behandlungsstrategien und Interventionen lassen sich auf dieser Grundlage gezielt analysieren. In der Versorgungspraxis können damit letztlich Behandlungsstandards verbessert, bestmögliche Versorgungsstrategien der Patient:innen unterstützt und das Arzt-Patienten-Verhältnis gestärkt werden.

Analysen zu Long COVID auf Basis von Sekundärdaten hätten beispielsweise das Potenzial, Erkenntnisse über erfolgversprechende oder ggf. kontraindizierte Therapiemaßnahmen zu identifizieren. Während inzwischen bekannt ist, dass Long COVID symptomatisch zahlreiche Ähnlichkeiten zu weiteren, wenig erforschten Erkrankungen wie dem chronischen Fatigue-Syndrom (ME/CFS) [5] oder dem Mastzellaktivierungssyndrom (MCAS) [6] aufweist, sind konkrete Therapiemöglichkeiten weiterhin rar. Entsprechend groß ist der Druck von Versorger:innen auf die Wissenschaft, Erkenntnisse zu liefern, um Betroffenen evidenzbasiert helfen zu können. Wenngleich eine Analyse von Abrechnungsdaten wahrscheinlich nicht sofort zu einer Therapieempfehlung führen wird, werden Analysen des Versorgungsgeschehens, verordneter Heil- und Hilfsmittel sowie longitudinale Beobachtungsstudien wertvolle Erkenntnisse liefern können.

Forschende können zukünftig gemäß § 8 Datentransparenzverordnung (DaTraV) [7] mit einem Antrag beim FDZ Gesundheit Zugriff auf einen Datenzuschnitt erhalten, der den Anforderungen ihrer Forschungsfrage entspricht. Innerhalb kurzer Zeit können sie dann den Programmcode für ihre Analysen entwickeln, um die benötigten statistischen Ergebnisse zu erhalten.

## Aufbau des Forschungsdatenzentrums Gesundheit

Damit die von den Krankenkassen erfassten Gesundheitsdaten aller rund 74 Mio. gesetzlich versicherten Menschen in Deutschland für Forschungszwecke schneller bereitgestellt und verwendet werden können, wird das FDZ Gesundheit als zentraler Ansprechpartner für alle mit Forschungsinteresse zur Nutzung dieser Sekundärdaten aufgebaut. Mit dem FDZ Gesundheit wird ein datenschutzkonformes, sicheres und dabei zugleich möglichst nutzungsfreundliches Portal errichtet, in dem unterschiedliche Prozesse wie Antragstellung, Datenbereitstellung, Datenanalyse und Ergebnisbereitstellung gebündelt abgebildet werden.

### Künftig verfügbare Daten

Das FDZ Gesundheit soll künftig mit den Krankenkassenabrechnungsdaten und den Daten der elektronischen Patientenakten nach § 363 SGB V [8] eine umfangreiche Datenbasis zur Verfügung stellen, die für die Forschung zu medizinischen Fragestellungen wie etwa zu Long COVID von großer Bedeutung ist. Neben ICD-codierten Diagnosen und OPS-codierten Prozeduren wird der Datenkranz der Krankenkassenabrechnungsdaten des FDZ Gesundheit auch Informationen zu Krankenhausaufenthalten, durchgeführten Operationen, verschriebenen Medikamenten und Facharztbesuchen umfassen. Die Daten, die allen nutzungsberechtigten Personen über das FDZ Gesundheit gemäß §§ 303a bis 303f SGB V [8] auf Antrag zur Verfügung gestellt werden können, sind in der Datentransparenzverordnung DaTraV § 3 „Art und Umfang der zu übermittelnden Daten“ [7] spezifiziert (vgl. Tab. 1). Potenzielle Datennutzende können aus verschiedenen Bereichen stammen, wie beispielsweise der Patientenvertretung, Organisationen der Leistungserbringenden, Forschungseinrichtungen oder aus der Gesundheitsberichterstattung. Der aktuelle Entwurf des Gesundheitsdatennutzungsgesetzes (GDNG) könnte im Bereich der Nutzungsberechtigung jedoch noch Änderungen bringen, die einen Zweckbezug statt eines Akteursbezugs vorsehen [9].

**Table 1:** 

Sektor	Datenfelder (beispielhaft)
Stammdaten	Geburtsjahr
	Geschlecht
	Betriebsnummer Krankenkasse
	Versichertentage (DMP, etc.)
	Versichertenstatus
	Versicherungsverhältnis
	Vitalstatus
	Sterbedatum
	Postleitzahl des Wohnorts
Ambulanter Sektor	Arztnummer (pseudonymisiert)
	Betriebsstättennummer (pseudonymisiert)
	Art der Behandlung, Beginn, Ende
	Art der Inanspruchnahme
	Entbindungsdatum
	Fallkosten inkl. Dialysesachkosten
	Zahnarztbefund mit Gebührenpositionen, Datum
	Erkrankungs- und Leistungsbereich nach § 116b
	Diagnosen (ICD), Art, Lokalisation, Diagnosesicherheit, Datum
	Prozeduren (OPS), Lokalisation, Datum
	Angaben zur Zweitmeinung
	Angaben der Terminservicestelle
	Bewertung und Vertragsnummer nach § 73b, § 140a, § 73c
	Integrierte und hausarztzentrierte Versorgung
Stationärer Sektor	Institutionskennzeichen (pseudonymisiert)
	Aufnahme- und Entlassungsdatum sowie -grund
	Datum der Behandlung
	Angaben zur Doppeluntersuchung
	Art der Belegleistung
	Diagnosen: Haupt- und primäre sowie sekundäre Neben‑, Aufnahme- und Entlassungsdiagnosen (ICD), Lokalisation, Diagnoseart
	Leistungsart, Leistungsschlüssel, Datum
	Prozeduren (OPS), Lokalisation, Datum
	Diagnosebezogene Fallgruppen (DRGs)
	Vor- und nachstationäre Pflegeleistungen sowie Entgelte
	Beatmungsstunden
	Arztnummer (pseudonymisiert) einweisender Arzt
	Institutionskennzeichen bei Verlegung oder Notfallaufnahme
Arzneimittelsektor	Verordnung, Verordnungsdatum, Dosierung, Bruttobetrag
	Arztnummer (pseudonymisiert)
	Betriebsstättennummer (pseudonymisiert)
	Pharmazentralnummer inkl. Sonderkennzeichen
	Institutionskennzeichen (pseudonymisiert) abgebende Apotheke
	Abgabedatum der Apotheke
	Kennzeichen Begründungspflicht für zahnärztliche Versorgung
	Vertragskennzeichen für einzelvertragliche Vereinbarungen zwischen Krankenkassen und Leistungserbringern
	Kennzeichen Sitz der Apotheke In- oder Ausland
	Mengenfaktor laut Verordnung je Position
	Verwendeter/abgegebener Anteil der Verpackung, Verwurf
	Vermerke wie Noctu-Kennzeichen, Aut-Idem
	Gesetzl. Abschläge, rezeptbezogene Zuzahlung, Eigenbeteiligungen
Weitere Sektoren, die planmäßig später hinzukommen sollen mit diversen Datenmerkmalen zu Heil- und Hilfsmitteln, Krankentransportleistungen, häuslicher Krankenpflege, Hebammenhilfe und digitalen Gesundheitsanwendungen	Abrechnungscode
	Tarifkennzeichen
	Positionsnummer der erbrachten Einzelleistung oder abgerechneten Leistung mit Anzahl, Menge, Einzelbetrag der Abrechnungsposition
	Betrag der gesetzl. Zuzahlung
	Eigenanteil und Mehrkosten
	Betriebsstättennummer (pseudonymisiert) des verordnenden Arzte
	Arztnummer (pseudonymisiert)
	Institutionskennzeichen (pseudonymisiert) des Leistungserbringers oder Krankenhauses
	Datum Erbringung und Verordnung
	Behandlungsbeginn und -ende
	Kilometerangabe
	Diagnose- oder Indikationsschlüssel
	Anwendungsort
	Geburtsdatum des Kindes oder der errechnete Geburtstermin bei vorgeburtlichen Leistungen
	etc.

*DMP* Disease-Management-Programm, *ICD* Internationale statistische Klassifikation der Krankheiten und verwandter Gesundheitsprobleme, *OPS* Operationen- und Prozedurenschlüssel

Analysen auf der Grundlage von Abrechnungsdaten der Krankenkassen werden bereits seit über 30 Jahren durchgeführt. Dabei gab es bisher einerseits die Möglichkeit, Daten bei einzelnen Krankenkassen zu beantragen. Diese Option steht nach § 75 SGB X [10] auch weiterhin zur Verfügung. Andererseits waren über eingesandte Skripte an das ehemalige Deutsche Institut für Medizinische Dokumentation und Information (DIMDI) Analysen auf Basis der Abrechnungsdaten gesetzlich versicherter Menschen in Deutschland möglich. Diese Daten stehen künftig auch im FDZ Gesundheit zur Verfügung und werden durch weitere Datenjahre und einen deutlich erweiterten Datenkranz ergänzt.

Die Einbeziehung dieser variablen Daten ermöglicht es Forschenden, sektorübergreifende Auswertungen durchzuführen und ein umfassendes Bild selbst von komplexen Erkrankungen wie Long COVID zu erhalten, die häufig Diagnostiken und Behandlungen im ambulanten und stationären Bereich erfordern. Besonders für Forschungen zu seltenen Erkrankungen bietet der enorm große Datensatz von allen 74 Mio. gesetzlich Versicherten in Deutschland große Chancen und Möglichkeiten, die in der Form bislang in Deutschland nicht möglich waren.

Bevor die Abrechnungsdaten beim FDZ Gesundheit ankommen, durchlaufen sie eine Reihe von Verarbeitungsschritten, um bereits in einem frühen Stadium eine Re-Identifikation einzelner Personen zu vermeiden:

Zunächst erfolgt die Dokumentation der Daten durch Ärzt:innen, Apotheken und andere Leistungserbringende im Gesundheitswesen. Diese Informationen werden dann zur Abrechnung an die jeweilige Krankenkasse weitergeleitet, die somit das medizinische Versorgungsgeschehen nahezu lückenlos abbilden.

**Figure 1:**
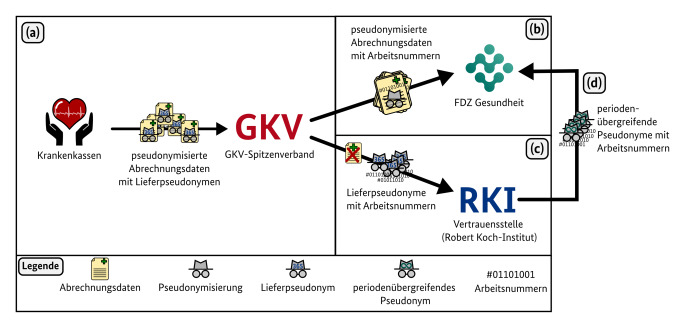

Nachdem die Daten bei den Krankenkassen eingegangen sind, gelangen sie in regelmäßigem Turnus gesammelt in bereits pseudonymisierter Form (mit sogenannten Lieferpseudonymen) zum Spitzenverband Bund der Krankenkassen (GKV-SV) (Abb. 1a). Auch Angaben über die Leistungserbringenden wie beispielsweise die „Lebenslange Arztnummer“ gelangen ausschließlich pseudonymisiert in den Datenbestand des FDZ. Auf diese Weise kann sowohl für die Versicherten als auch für die Versorger:innen der Datenschutz gewährleistet werden, während zugleich sinnvolle Analysen für die Patientenversorgung möglich sind. Einzelne notwendige Angaben wie beispielsweise Informationen über die Fachgruppe der Versorger:innen bleiben erhalten.

Die pseudonymisierten Daten werden nach einer ersten Qualitätskontrolle vom GKV-SV zusammen mit einer durch den GKV-SV zu Zuordnungszwecken vergebenen „Arbeitsnummer“ verschlüsselt an das FDZ Gesundheit übermittelt (Abb. 1b). Gleichzeitig werden die Arbeitsnummern, die u. a. für die Zuordnung zum „periodenübergreifenden Pseudonym“ der Vertrauensstelle notwendig sind, zusammen mit den Lieferpseudonymen (aber ohne medizinische Daten) an die Vertrauensstelle beim Robert Koch-Institut (RKI) geschickt (Abb. 1c).

Das RKI stellt daraufhin die „periodenübergreifenden Pseudonyme“ bereit, die eine sichere Verknüpfung der Daten über mehrere Jahre hinweg ermöglichen. Diese periodenübergreifenden Pseudonyme gelangen zusammen mit der Arbeitsnummer anschließend zum FDZ Gesundheit (Abb. 1d), sodass die Datenbestände verschiedener Jahre harmonisiert werden können. Auf diese Weise sind auch longitudinale, versichertenbezogene Auswertungen über mehrere Abrechnungsjahre hinweg möglich, während gleichzeitig der Datenschutz weiterhin gewährleistet ist. Gerade im Fall von Long COVID hat dies zum Beispiel das Potenzial, Langzeitfolgen über viele Jahre hinweg zu untersuchen.

### Antragsprozess

Damit Forschende einen Antrag auf die Nutzung der Gesundheitsdaten zu Analysezwecken stellen können, wird zunächst die Online-Registrierung ihrer Institution im Portal des FDZ notwendig sein. An dieser Stelle ist eine Authentifizierung der Person notwendig, die für das geplante Forschungsprojekt verantwortlich ist. Sofern eine Nutzungsberechtigung nach § 303e Absatz 1 SGB V der DaTraV vorliegt, wird die Institution durch Mitarbeitende des FDZ freigeschaltet. Ab diesem Zeitpunkt ist eine Antragstellung auf Datennutzung möglich.

Der vollständig digital zu erfassende Antrag muss dabei unter anderem Angaben zum Zweck des Forschungsvorhabens, den geplanten Auswertungsmethoden sowie über die benötigten Daten enthalten. Auch die an der Analyse beteiligten Personen sind dabei anzugeben. Im Falle des Beispiels der Long-COVID-Analysen könnte also z. B. angegeben werden, dass es sich um ein Forschungsvorhaben zum Zweck der Verbesserung der Versorgungsqualität oder um Analysen des Versorgungsgeschehens handelt. Dabei könnten exemplarisch Daten ab dem Jahr 2019, also dem Beginn der Pandemie, benötigt werden, die u. a. Angaben zu Geschlecht, Alter und ICD-Nummern enthalten. Auf diese Weise könnte eine geschlechts- und altersabhängige Auswertung unter Berücksichtigung der Long-COVID-Diagnose und ggf. weiterer Erkrankungen durchgeführt werden.

Das FDZ Gesundheit sorgt bereits in der aktuellen Aufbauphase dafür, dass viele Prozesse im späteren Betrieb automatisiert und damit skalierbar ablaufen.

Der Antragsprozess beim FDZ Gesundheit wird im Vergleich zu bisherigen Möglichkeiten effizient und zeitsparend erfolgen können. Forschende brauchen nach erfolgter Registrierung lediglich einen digitalen Antrag zu stellen und erhalten bei positiver Beurteilung anschließend Zugriff auf einen i. d. R. anonymisierten, in begründeten Einzelfällen pseudonymisierten Zuschnitt der gewünschten Daten in einem eigens zu diesem Zweck erzeugten virtuellen Analyseraum. Dieser wird im nachfolgenden Abschnitt näher erläutert. Der Rest des Prozesses geschieht im Hintergrund durch die Mitarbeitenden des FDZ, die den Antrag überprüfen und über die Freigabe entscheiden. Die Kriterien, die dieser Entscheidung zugrunde liegen, werden auf der Website des FDZ veröffentlicht, sodass eine transparente Entscheidungsfindung gewährleistet ist. Dies ermöglicht es den Wissenschaftler:innen, schnell mit der Analyse zu beginnen und ihre Forschung voranzutreiben. Im Vergleich zu einer oder mehreren Anfragen bei einzelnen Krankenkassen zur „Übermittlung von Sozialdaten für Forschung und Planung“ nach § 75 SGB X [10] spart dieser zentralisierte Antragsprozess viel Zeit und Aufwand – sowohl für die Forschenden, als auch für die jeweiligen Krankenkassen.

### Datenbereitstellung

Das FDZ Gesundheit stellt die angeforderten Daten in einer sicheren und geschützten Umgebung bereit. Forscher:innen können die Daten in einer ausschließlich für sie bereitgestellten virtuellen Analyseumgebungen auswerten.

Die bereitgestellten Abrechnungsdaten der Krankenkassen werden im FDZ Gesundheit in einem zusätzlich gesicherten und für Forscher:innen unzugänglichen Bereich (isolierte Zone) in einer Datenbank gespeichert. In dieser Zone liegen die Daten in pseudonymisierter Form und mit dem „periodenübergreifenden Pseudonym“ verknüpft vor. Die Pseudonymisierung mit periodenübergreifendem Pseudonym sowie einer Zuordnungsnummer ermöglichen sowohl longitudinale, versichertenbezogene Auswertungen als auch die Rückverfolgung von Krankenkassenwechslern. Externe Personen erhalten zu keinem Zeitpunkt Zugriff auf die Daten in der isolierten Zone.

Die tatsächliche Datenanalyse findet ausschließlich in einem Analyseraum statt, der spezifisch für den bewilligten Antrag durch das FDZ angelegt wird. Die virtuellen Analyseräume sind sichere Umgebungen, die beispielsweise einen Import oder Export von und nach außen verhindern. Dies gewährleistet ein Höchstmaß an Sicherheit und Datenschutz. Forscher:innen haben hier die Möglichkeit, ihre Analysen durchzuführen und statistische Ergebnisse zu generieren, ohne direkten Zugriff auf die vollständigen Originaldaten zu haben. Für das bewilligte Forschungsvorhaben wird durch die Mitarbeitenden des FDZ ein Datenzuschnitt erstellt, auf dem die Forschenden ihre Auswertungsskripte entwickeln und testen können. Dieser Datenzuschnitt enthält die zum Testen der Auswertungsskripte erforderlichen Tabellenspalten, die für die Analyse des Forschungsvorhabens beantragt wurden und notwendig sind. Die Anonymisierungsverfahren, die im Falle eines Antrags auf anonymisierte Daten Anwendung finden, werden derzeit noch festgelegt.

Der für das jeweilige Forschungsprojekt individuelle Datenzuschnitt dient den Forscher:innen dazu, ihre Auswertungsskripte zu erstellen; die finalen Ergebnisse basieren auf der Gesamtheit der originalen Abrechnungsdaten. Ihr Skript können die Forscher:innen dazu über das Portal des FDZ einreichen und darauf basierend Zwischenergebnisse oder auch ein finales Ergebnis anfordern. Die Analyseskripte werden nach einer Sicherheitsüberprüfung auf Basis der (pseudonymisierten) Originaldaten in einer isolierten und für Forschende jederzeit unzugänglichen Umgebung ausgeführt. Es entsteht eine Ergebnismenge mit aggregierten Daten, die zunächst von Mitarbeitenden des FDZ hinsichtlich eines möglichen Re-Identifikationsrisikos überprüft und bewertet wird. An dieser Stelle können gegebenenfalls iterative Anpassungen notwendig sein, bevor die Ergebnismenge an die Forschenden übermittelt werden kann.

Um Auswertungen auch weniger programmieraffinen Forscher:innen zu erleichtern, sollen zukünftig zwei weitere Alternativen zur eigenen Skriptentwicklung möglich sein: Ein Point-and-click-Tool sowie vordefinierte Standardauswertungen. Beide Erweiterungen dienen der Vereinfachung von Analysen und der Unterstützung der Forschenden. Mit dem Point-and-click-Tool wird es möglich sein, unterschiedliche statistische und epidemiologische Maßzahlen wie beispielsweise eine Inzidenz oder eine Prävalenz durch direkte Auswahl berechnen zu lassen. Bei den geplanten Standardauswertungen handelt es sich um komplexere vordefinierte Skripte, die spezifische Ergebnismengen liefern. Ziel dieser Auswertungen ist es, häufig vorkommende Analysen von hohem Interesse abzubilden, sodass die Forschenden einen Grundstock an vordefinierten Analysemodulen direkt verwenden können. Einige erste geeignete Standardauswertungen werden derzeit im ReFern-Projekt gemeinsam mit dem RKI entwickelt (siehe Beitrag von Krause et al. in diesem Themenheft).

Im Beispiel der Long-COVID-Analyse könnte die Ergebnismenge je nach spezifischer Forschungsfrage beispielsweise statistische Ergebnisse über Häufigkeiten von Vorerkrankungen Betroffener ggf. stratifiziert nach Altersgruppen und Geschlecht etc. enthalten. Auch Ergebnistabellen zu Häufigkeiten von bestimmten Prozeduren oder Verschreibungen von bestimmten Medikamenten im Rahmen der Therapie sind denkbar.

### Sicherheitsaspekte und Design-Prinzipien

Unsere Gesundheitsdaten sind sensiblen Daten und bedürfen höchster Sorgfalt. Eine Minimierung des Risikos zur Re-Identifizierung einer Person ist daher von großer Bedeutung und nach § 303d SGB X gesetzliche Aufgabe des FDZ Gesundheit [10]. Entsprechend gelten für das FDZ sehr hohe Anforderungen an das Datenschutz- und das IT-Sicherheitsniveau.

Im Hinblick auf die Sicherheit der Daten werden im FDZ Gesundheit mehrere Maßnahmen ergriffen. Zum einen finden sämtliche Analysen ausschließlich in einer gesicherten virtuellen Analyseumgebung statt. Die strikte Trennung zwischen der isolierten Zone und den Analyseräumen im FDZ Gesundheit stellt sicher, dass die sensiblen Gesundheitsdaten geschützt bleiben und je nach Forschungsfrage i. d. R. nur anonymisierte, mindestens jedoch pseudonymisierte Datenzuschnitte unter strengen Sicherheitsvorkehrungen analysiert werden können. Dieses Sicherheitskonzept sorgt dafür, dass die Privatsphäre der Versicherten gewahrt bleibt und keine unautorisierte Nutzung der Daten stattfindet.

Weiterhin erfolgt die Übermittlung der Ergebnisse ausschließlich nach einer Überprüfung der Ergebnismenge und in aggregierter Form, um das Re-Identifikationsrisiko noch weiter zu minimieren. Einzelfalldaten können zu keinem Zeitpunkt aus dem System exportiert werden. Auf diese Weise findet eine zweifache Datenminimierung statt: Während die zur Skriptentwicklung im Analyseraum bereitgestellten Daten bereits auf eine für die Forschungsfrage notwendige Menge reduziert werden und im Regelfall anonymisiert vorliegen, werden zusätzlich die finalen Ergebnisse aggregiert herausgegeben. Anstatt individueller Daten werden also nur zusammengefasste Informationen für die Verwendung außerhalb der Analyseräume (also z. B. für die Publikation in wissenschaftlichen Journals) zur Verfügung gestellt. Insgesamt werden so die Informationen auf ein geeignetes Maß reduziert, das einerseits die Durchführung von aussagekräftigen Analysen erlaubt, während gleichzeitig das Recht auf informationelle Selbstbestimmung der Versicherten geschützt wird. Für Forschende, die beispielsweise Erkenntnisse zu Long COVID gewinnen möchten, ist dieser Balanceakt zwischen Forschungsbedarf und Datenschutz von großer Bedeutung. Auf diese Weise haben sie die Möglichkeit, in einer geschützten Umgebung Analysen durchzuführen und statistische Ergebnisse für wichtige wissenschaftliche Publikationen zu generieren, während gleichzeitig die Gesundheitsdaten der Versicherten sicher geschützt bleiben. Persönliche Informationen von Versicherten werden nicht preisgegeben.

### Datenlinkage

Erklärtes Ziel eines Datenlinkage ist es, „dass die Daten einer Person aus zwei Datenquellen fehlerfrei zusammengeführt werden“ [11]. Auf diese Weise lassen sich eine zusätzliche Validierung beider Datenquellen sowie umfangreichere Analysen ermöglichen [12]. Aus den Reihen der späteren Nutzer:innenschaft des FDZ wurde der Wunsch geäußert, im Rahmen der jeweils aktuellen Gesetzeslage Synergien aus unterschiedlichen Datenquellen innerhalb der FDZ-Analyseräume nutzen zu können [13]. Die Voraussetzungen für geeignete Verlinkungen zwischen unterschiedlichen Datenquellen sind konzeptionell bereits angelegt, da die Abrechnungsdaten, wie oben beschrieben, unter Einbezug der Vertrauensstelle bereits über die Jahre hinweg verknüpfbar gemacht werden. Das gleiche Prinzip ist im Verlauf auch für die Verknüpfung zwischen den Abrechnungsdaten und den Daten aus der elektronischen Patientenakte (ePA) gemäß § 363 SGB V vorgesehen [8]. Zum jetzigen Zeitpunkt ist ein Datenlinkage mit externen Datenquellen noch nicht möglich, jedoch im aktuellen Entwurf des Gesundheitsdatennutzungsgesetzes [9] vorgesehen.

Eine mögliche zukünftige Verknüpfung mit den Daten der elektronischen Patientenakte (ePA) bietet das Potenzial, die Analyse der Sekundärdaten durch weitere RWD anzureichern. Durch die Verknüpfung dieser Datenquellen ergeben sich neue Möglichkeiten für die unterschiedlichsten medizinischen Forschungsfragen. Die ePA bietet zusätzliche Informationen, die für die Analyse von Langzeitfolgen und Therapieansätzen von großer Bedeutung sein können. In unserem Beispiel für Long-COVID-Analysen könnte beispielsweise der elektronische Impfpass wertvoll sein, der genaue Auskünfte über die Zeitpunkte der COVID-Impfungen, das verwendete Vakzin etc. geben könnte, sofern die Angaben aus dem papierbasierten Impfpass nachträglich eingepflegt werden, was auch für die Nutzung der ePA in der Versorgungssituation wichtig wäre. Aus dem elektronischen Mutterpass könnten im Falle eines Nachtrags der Daten wichtige Informationen über Komplikationen im Rahmen einer Schwangerschaft bei Long-COVID-Erkrankten hervorgehen.

Auch Verknüpfungen der im FDZ vorliegenden Daten mit anderen wichtigen Datenquellen bieten für die Wissenschaft großes Potenzial. Beispielsweise könnte ein Linkage mit Daten der Krebsregister, wie es im GDNG-Entwurf vorgesehen ist, ermöglichen, Krebserkrankungen sowohl sektorübergreifend und longitudinal als auch mit tiefergehenden Informationen wie Tumorstadien, Histologie und genetischen Varianten zu untersuchen [14]. Damit bei einem Linkage der Bezug zwischen den Daten sichergestellt werden kann, treffen diese Vorteile in der Form nur auf solche Quellen zu, die passend zu den Daten des FDZ pseudonymisiert werden können.

## Fazit

Insgesamt eröffnet das FDZ Gesundheit neue Möglichkeiten für die medizinische Forschung, eine verbesserte Versorgung und Erkenntnisgewinn für die Gesundheit der Bevölkerung. Es fördert die evidenzbasierte Medizin und ermöglicht die Nutzung umfangreicher Real-world-Daten, was der Wissenschaft, der Gesundheitsversorgung und der gesamten Gesellschaft zugutekommt. Wenn die bekannten Limitationen der Sekundärdaten beachtet werden, werden die mit ihnen durchgeführten Analysen klinische Studien ganz entscheidend ergänzen können. Dabei erfüllt das FDZ Gesundheit Anforderungen zur Sicherheit, gerade im Hinblick auf den notwendigen Datenschutz der sensiblen Gesundheitsdaten, und deckt den Bedarf der Forschenden, geeignete Analysen durchführen zu können.

## Footnotes

1 https://www.forschungsdatenzentrum-gesundheit.de (englischsprachige Website: https://www.healthdatalab.de/).

2 https://www.bundesgesundheitsministerium.de/presse/pressemitteilungen/bmg-initiative-long-covid-pm-12-07-23.html (Zugriff: 13.07.2023).
